# Dietary Inflammatory Index and Cardiometabolic Risk Parameters in Overweight and Sedentary Subjects

**DOI:** 10.3390/ijerph14101104

**Published:** 2017-10-06

**Authors:** Claudia Marcela Camargo-Ramos, Jorge Enrique Correa-Bautista, María Correa-Rodríguez, Robinson Ramírez-Vélez

**Affiliations:** 1 Centro de Estudios para la Medición de la Actividad Física (CEMA), Escuela de Medicina y Ciencias de la Salud, Universidad del Rosario, Bogotá DC 111221, Colombia; camargoclaudia88@gmail.com (C.M.C.-R); jorge.correa@urosario.edu.co (J.E.C.-B.); 2 Departamento de Enfermería, Facultad de Ciencias de la Salud Avda, De la Ilustración, s/n, (18016), Universidad de Granada, Granada 18071, Spain; macoro@ugr.es

**Keywords:** dietary inflammatory index, cardio-metabolic, diet, overweight

## Abstract

Nutrition has been established as a relevant factor in the development of cardiovascular disease (CVD). We aimed to investigate the relationship between the dietary inflammatory index (DII) and cardiometabolic risk parameters in a cohort of 90 overweight and sedentary adults from Bogotá, Colombia. A 24-h dietary record was used to calculate the DII. Body composition variables, flow-mediated dilation (FMD), pulse wave velocity (PWV), lipid profile, glucose, glycosylated hemoglobin (Hb1Ac), and blood pressure were measured and a cardiometabolic risk score (MetScore) was calculated. A lower DII score (anti-inflammatory diet) was significantly associated with higher high-density lipoprotein-cholesterol (HDL-C) and FMD, and lower Hb1Ac and MetScore (*p* < 0.05). A lower DII score was inversely correlated with plasma triglyceride levels (*r* = −0.354, *p* < 0.05), glucose (*r* = −0.422, *p* < 0.05), MetScore (*r* = −0.228, *p* < 0.05), and PWV (*r* = −0.437, *p* < 0.05), and positively with FMD (*r* = 0.261, *p* < 0.05). In contrast, a higher DII score (pro-inflammatory diet) showed a positive relationship with MetScore (*r* = 0.410, *p* < 0.05) and a negative relationship with FMD (*r* = −0.233, *p* < 0.05). An increased inflammatory potential of diet was inversely associated with an improved cardiometabolic profile, suggesting the importance of promoting anti-inflammatory diets as an effective strategy for preventing CVD.

## 1. Introduction

Low-grade chronic inflammation has been established as a relevant factor in the development of cardiovascular disease (CVD) [1]. It is characterized by increased circulating levels of cytokines with inflammatory activity and acute-phase proteins (C-Reactive Protein; CRP), interleukins (IL-1, IL-6), and tumor necrosis factor alpha (TNF-α), among others—as well as by increased infiltration of macrophages in peripheral tissue [2]. Evidence has shown that lifestyles, especially dietary factors, may modulate this process [3,4,5]. Thus, nutrition plays an important role in the development of atherogenesis and cardiovascular events [3,6].

In this context, it has been indicated that dietary patterns characterized by a high intake of saturated and trans unsaturated fatty acids may lead to alterations in endothelial function [7,8]. The “Western” diet, rich in red meats, fats, and carbohydrates, has been positively associated with markers of low-grade subclinical inflammation, while dietary patterns based on fruits, vegetables, olive oil, and whole grains have shown an inverse relationship [3,4,9]. In addition, the Mediterranean food pattern, which is a well-known anti-inflammatory diet, exerts a protective effect against free radicals and oxidants [10].

A dietary pattern comprising high amounts of fish, yogurt, vegetables, pasta, greens, fruit, and wine also has the potential to reduce circulating levels of inflammatory markers [3]. Diet-based anti-inflammatory components, including omega-3 fatty acids, vitamins B1 (thiamine), B2 (riboflavin) and B3 (niacin), folic acid, vitamin A, vitamin C, vitamin E, beta-carotene, magnesium, and zinc, have been identified [11].

On this basis, researchers from the University of South Carolina in the United States designed the dietary inflammatory index (DII), a tool for assessing the inflammatory diet profile based on 45 nutritional parameters. Due to their anti-inflammatory or pro-inflammatory properties, these nutritional factors were associated with markers of inflammation including CRP, interleukins IL-1β, IL-4, IL-6 and IL-10, and TNF-α [12]. The DII has been reported to be associated with several inflammatory processes, such as obesity, insulin resistance, cardiovascular risk, and several types of cancer [13,14,15,16]. However, to the best of our knowledge, the DII has not been used to evaluate inflammatory properties of diet in Latin Americans, characterized by increasing total fat, animal products, and sugar intakes [14], particularly in the Colombian population. Since Latin Americans have different dietary habits and morbidity and mortality indicators, it is of particular interest.

Recently, there has been a surge of interest in developing strategies with a more cost-effective approach and fewer side effects, which is useful in clinical practice aimed at preventing CVD. Among the non-pharmacological strategies, physical activity and diet are considered the cornerstones of cardiovascular prevention. Taking all this evidence into account, we aimed to investigate the relationship between the DII and cardiometabolic risk parameters in a cohort of overweight and sedentary adults from Bogotá, Colombia.

Our hypothesis is that higher DII scores (pro-inflammatory diet defined by positive values) are associated with an increased cardiometabolic risk profile, independently of other risk factors such as age and gender.

## 2. Materials and Methods

### 2.1. Study Design

A secondary analysis of the clinical trial High Intensity Interval- vs. Resistance or Combined- Training to Improve Cardiometabolic Health in Overweight Adults: Cardiometabolic HIIT-RT Study ClinicalTrials.gov Identifier: NTC02715063 was carried out between 2016 and 2017 [17]. The study was performed in accordance with the Declaration of Helsinki (2000) and was approved by the Human Ethics Committee of Manuela Beltrán University (Code N° 06-1006-2014; Resolution 008430/2003 by the Colombian Ministry of Health). Written informed consent was obtained from each participant.

### 2.2. Participants

The sample comprised 90 women and men, with excess body weight (BMI ≥ 26 and ≤ 35 kg/m^2^), who were sedentary (individuals who do not engage in five or more days of moderate physical activity or walking for at least 30 min per session, or who do not engage in three or more days per week of vigorous physical activity for 20 min, as assessed by the International Physical Activity Questionnaire (IPAQ) [18], and who met at least one of the criteria of metabolic syndrome as established by the International Diabetes Federation [18]. The study cohort was selected through a call for volunteers. Exclusion criteria included medical diagnosis of hypertension, hypo/hyperthyroidism, history of alcohol and drug abuse, use of lipid-lowering medications, or drugs capable of modifying the lipid profile four weeks before the study, following hypocaloric diets to lose weight and suffering from inflammatory or infectious processes.

### 2.3. Procedures

Participants completed questionnaires regarding medical history including personal and family history of CVD, smoking status (number of cigarettes/day), alcohol consumption (grams of alcohol/day), physical activity (min/week), and medication use.

Anthropometric measurements, including height, weight, waist circumference (WC), and hip circumference (HC), were evaluated according to standardized protocols for the Colombian population established by the World Health Organization (WHO) [19]. Height was measured to the nearest 0.1 cm using a portable stadiometer (SECA 206^®^, range 0–220 cm, Seca GmbH &. Co, Hamburg, Germany). To measure weight, we used an electronic scale (Seca mBCA 515^®^ HANS E. RÜTH S.A, Allers Co, Hamburg, Germany). Body mass index (BMI) was calculated as weight over height squared (kg/m^2^). An anthropometric a metal tape (Lufkin W606PM^®^, Allers Co, Parsippany, NJ, USA), with an accuracy of ± 1 mm was used to measure hip circumferences according to anatomical references established by the WHO [20]. Body composition parameters, including fat and lean mass in grams and percentage (trunk, gynecoid, android, android/gynecoid (A/G) fat quotient) and total tissue, were measured by dual-energy X-ray absorptiometry (DXA, Hologic Mod Explorer^®^, 4500 C/W QDR, INC 35 Crosby Drive, Bedford, USA), and the regions of interest (ROIs) were selected. These measurements were performed after 10–12 h of fasting.

Blood pressure was taken with an automatic monitor (Omrom^®^ HEM 705 CP, Health-care Co, Kyoto, Japan) following the recommendations of the European Heart Society (on the right arm, with participants in a supine position and after 10 min of rest). Mean arterial pressure (MAP) was calculated using the following formula: (2 (systolic blood pressure (SBP) + diastolic blood pressure (DBP))/3).

Capillary blood samples (40 µL) were collected for determining serum biochemical parameters, including fasting glucose, triglycerides (TG), high-density lipoprotein-cholesterol (HDL-C), and total cholesterol (TC) using portable Cardiocheck^®^ equipment (Mexglobal SA, Parsippany, NJ, USA). Low-density lipoprotein-cholesterol (LDL-c) was calculated using Friedewald’s Formula when triglyceride values were ≤400 mg/Dl [21]. Glycated hemoglobin (Hb1Ac) was measured using A1cNow + (Bayer^®^, Mexglobal SA, Terrytown, NY, USA). Blood samples were drawn between 07:00 and 09:00, after 10–12 h of fasting (11.2 h on average). A cardiometabolic risk score (MetScore) was calculated as the sum of the typified Z-scores per age and gender from the following components: WC, TG, HDL-C, glucose, and MAP [18]. MetScore = ((♂40 or ♀50 − HDL-C)/SD × (−1)) + ((triglycerides − 150)/SD) + ((glucose − 100)/SD) + ((WC − ♂90 or ♀80)/SD) + ((MAP − 100)/SD). Cut-off points to calculate the MetScore were determined according to the International Diabetes Federation criteria [18]. A MetScore standard deviation score ≥1 was considered to represent a high risk of CVD.

Brachial artery flow-mediated dilation (FMD) was used as a measure of endothelium-dependent vasodilation. The peripheral vessel ultrasound evaluation was performed with a high-resolution ultrasound system (Mindray M-9^®^, Invermedica LTDA, Shenzhen, China), with a 7.5 MHz linear array probe to locate and interrogate flow velocity profiles in the right brachial artery. Ultrasound images were obtained after 20 min of rest in a supine position in a dark, climate-controlled, quiet room (22–24 °C), with the participant’s arm immobilized and slightly supinated and elevated. An additional 10 min of rest was allowed before the opposite arm was imaged. The right arm was imaged first in each case. After a resting period of at least 20 min, 1 min of baseline recording of the brachial artery diameter was performed. Subsequently, the occlusion cuff was inflated to >200 mmHg for 5 min [22]. To estimate brachial artery shear stress, peak shear rate was calculated as follows: peak SR = maximal flow velocity (mm/s)/baseline diameter (mm). Normalized brachial artery FMD was calculated to account for the peak shear rate using the following equation: normalization of brachial artery flow-mediated dilation (FMDn) = brachial artery FMD/peak shear rate x 100. Brachial artery diameter recording was restarted at least 30 s before cuff deflation and continued for 3 min thereafter. Peak artery diameter and the time to reach this peak after cuff deflation were recorded. The intra-session coefficient of variations was ≤1% for the baseline diameter. Reliability, estimated by intra-class correlation coefficients (ICCs) based on four baseline measurements (*n* = 8 subjects), showed an ICC of 0.91 for baseline diameter and 0.83 for FMD.

Aortic pulse wave velocity (PWV) was measured with the oscillometric method using the occlusion technique [23] by Arteriograph (TensioMedTM software v 1.9.9.2, Mexglobal SA, Budapest, Hungry). A detailed description of the PWV technique can be found elsewhere [23]. Outcome measures were assessed by personnel blinded.

### 2.4. Dietary Inflammatory Index

The DII was calculated from 28 food parameters, including carbohydrates, proteins, fats, fiber, iron, zinc, magnesium, vitamin A, vitamin C, vitamin B6, vitamin B12, vitamin E, vitamin D, thiamine, riboflavin, niacin, selenium, folic acid, saturated fats, monounsaturated fats, polyunsaturated fats, omega 3 fatty acids, omega 6 fatty acids, trans-type fatty acids, β-carotene, and caffeine. Food consumption was estimated from a 24 h dietary record (mean between one weekday and one weekend day). Data were checked by a nutritionist, and standard household measures were used. DII scores were recoded as a pro-inflammatory diet (positive values) or anti-inflammatory diet (negative values) based on previous studies [24].

### 2.5. Statistical Analysis

All statistical analyses were performed using the Statistical Package for the Social Sciences® software, version 22 for Windows (SPSS^®^, Inc., IBM, Chicago, IL, USA). The Kolmogorov–Smirnov normality test was conducted before association analysis. Continuous variables were presented as mean ± standard deviation and categorical variables were presented as frequencies and percentages (*n* and %). The differences were estimated using Student’s *t*- or chi-square tests depending on the type of variable. DII scores were recoded as a pro-inflammatory diet (positive values) (≥2.03, min/max (0.13 to 3.64); *n* = 77) or anti-inflammatory diet (negative values) (≤ −1.26, min/max (−3.71 to −0.37); *n* = 13). Partial Pearson correlation coefficients between the DII and the cardiometabolic risk parameters adjusted for age and gender were performed. The significance level was set at *p* < 0.05. The DII score ranged between −3.71 and 3.64.

## 3. Results

**Figure 1:**
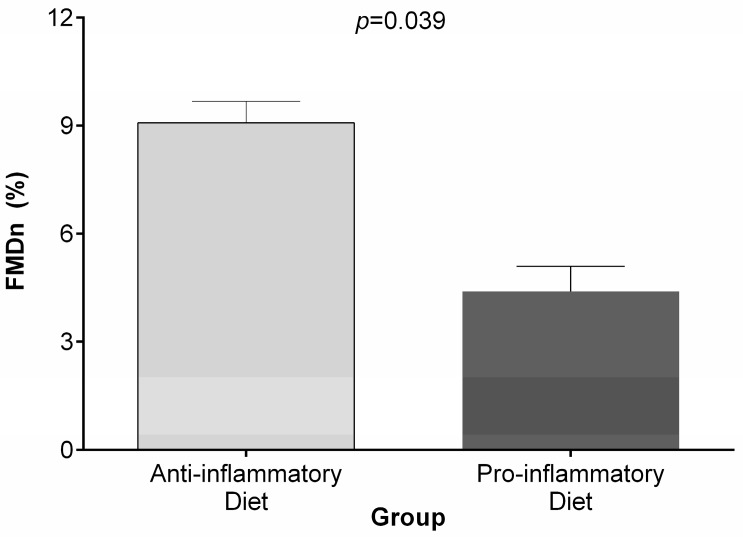
Differences among the categories of the dietary inflammatory index (DII) and normalized brachial artery flow-mediated dilation (FMD).

The anthropometric, body composition, metabolic markers, and endothelial function variables assessed in this study are shown according to the categories of the DII score in Table 1. The mean age of the study population was 39.7 ± 6.9 years and the mean BMI was 30.0 ± 3.5 kg/m^2^. Lower DII score (anti-inflammatory diet) was significantly associated with higher HDL-C (38.7 mg/dL ± 9.2) and FMD (12.3% ± 7.9) and lower Hb1Ac (5.3% ± 0.5) and MetScore (*p* < 0.05). For the anthropometric, body composition, and blood pressure measurements, no significant differences among the categories of the DII were identified. Statistically significant differences were identified between the categories of the DII and normalized brachial artery FMD (Figure 1).

Table 2 shows the macro- and micronutrients intakes according to the categories of the DII. Higher intakes of fiber, iron, zinc, magnesium, vitamin A, vitamin C, thiamine, riboflavin, niacin, omega 3, vitamin B6, folic acid, vitamin B12, beta-carotene, vitamin E, and vitamin C were observed in the anti-inflammatory category compared to the pro-inflammatory category (*p* < 0.05).

Table 3 shows partial correlations adjusted for age and gender. Lower DII score (anti-inflammatory diet) was inversely correlated with plasma triglyceride levels (*r* = −0.354, *p* < 0.05), glucose (*r* = −0.422, *p* < 0.05), MetScore (*r* = −0.228, *p* < 0.05), and PWV (*r* = −0.437, *p* < 0.05) and positively correlated with FMD (*r* = 0.261, *p* < 0.05). On the contrary, higher DII score (pro-inflammatory diet) showed a positive relationship with MetScore (*r* = 0.410, *p* < 0.05) and a negative relationship with FMD (*r* = −0.233, *p* < 0.05). Forty percent of the subjects had a MetScore ≥1, showing statistically significant differences among the categories of the DII categories. An anti-inflammatory diet was inversely correlated with percentage of fat tissue and total cholesterol values; however, these associations were not statistically significant.

## 4. Discussion

Adherence to a healthy dietary pattern has been postulated as a determinant factor in regulating inflammatory markers [6,24]. Despite previous research have reported that the DII was associated with several inflammatory processes including obesity or certain cancers, this study, the first of its kind, investigated the relationship between the DII and cardiometabolic risk parameters was investigated in a cohort of overweight/obese and sedentary adults from Bogotá, Colombia. Our data demonstrate that an anti-inflammatory diet was significantly associated with higher HDL-C and FMD values, as well as lower Hb1Ac and MetScore values, after adjusting for age and gender, thereby supporting the benefits of an anti-inflammatory diet in regard to parameters related to CVD.

To the best of our knowledge, this is the first study to evaluate the association between the DII and markers of endothelial function. Our results indicate that a more anti-inflammatory diet profile is associated with improved endothelial function, showing an inverse relationship with PWV and a positive relationship with FMD. These findings were in concordance with previous studies in which the association between healthy dietary patterns and parameters of endothelial function was investigated [8,10,25,26]. Rallidis et al. [25] demonstrated that close adherence to a Mediterranean diet in subjects with abdominal obesity increases FMD and decreases diastolic blood pressure (DBP) and insulin resistance (HOMA-IR). Similarly, the Mediterranean-style dietary pattern improved markers of endothelial function in patients with metabolic syndrome [26]. These data are in line with a recent meta-analysis that found that a Mediterranean diet reduces inflammation and improves endothelial function [10]. Furthermore, a lower intake of fiber, polyunsaturated fats, and vegetable protein and a higher intake of cholesterol were associated with increasing impairment of endothelial function in patients with type I diabetes [27]. Interestingly, a prospective study conducted by our research group in a Colombian population supported the contribution of postprandial lipemia to endothelial dysfunction [8].

With regard to conventional cardiovascular risk factors, we observed that an anti-inflammatory diet was related to higher HDL-C and lower Hb1Ac levels. Furthermore, correlation analysis identified that an increased anti-inflammatory potential of diet was inversely associated with plasma glucose and triglyceride levels. These results are in line with data from the Supplémentation en Vitamines et Minéraux AntioXydants (SU.VI.MAX) study conducted in a cohort of 3726 subjects, which demonstrated that a pro-inflammatory diet was associated with higher triglyceride and lower HDL-C levels [16]. Similarly, Sokol et al. reported an inverse relationship between the DII and HDL-C in a Polish population of 3862 participants [28]. Taking into account our association findings and the above lines of evidence, a pro-inflammatory diet might have a negative effect on the cardiometabolic risk profile across different populations [29,30,31,32].

On the other hand, this study identified an inverse association between an anti-inflammatory diet and the cardiometabolic risk score (MetScore). Previous works have explored the relationship between the DII and components of metabolic syndrome, yielding inconsistent conclusions [16,28,33]. In the SU.VI.MAX study, the odds of metabolic syndrome were 39% higher among subjects with a pro-inflammatory diet than in those with an anti-inflammatory diet [16]. Conversely, other authors have reported a lack of association between the DII and the MetScore [28,33]. It is interesting to note that in our study the prevalence of metabolic syndrome was 40%, whereas in the above-mentioned studies it was 30% and 28%, respectively. Thus, the contradictory results may be explained by the differences in the prevalence of metabolic syndrome across study cohorts.

The mechanisms by which an anti-inflammatory diet may regulate low-grade inflammation in cardiometabolic diseases are not certain. Nonetheless, it has been hypothesized that unhealthy dietary patterns with pro-inflammatory properties could trigger an innate immune response related to an increased production of pro-inflammatory cytokines and reduced production of anti-inflammatory cytokines, promoting states of chronic inflammation and consequently an increased risk of endothelial dysfunction, metabolic syndrome, and CVD [34,35].

In this study, we reported no significant differences in any anthropometric parameters or body composition variables among the categories of the DII. It is worth noting that we evaluated, for the first time, the possible relationship between the DII and measurements of body composition using a DXA device. Although the Prevención con Dieta Mediterránea (PREDIMED) study demonstrated significant associations between the DII and anthropometric data, only BMI, WC, and WHtR variables were investigated [15]. Moreover, the fact that the participants included in this study had similar anthropometric characteristics due to the inclusion criteria could explain the lack of significant associations reported in this study. Hence, further studies conducted in populations with heterogeneous anthropometric variables are needed to clarify this issue.

The present study has some limitations. First, due to its cross-sectional design, no causal conclusions can be drawn. Secondly, the DII was calculated from 24 h dietary records. Although the literature supports its use as a pertinent method for assessing nutrient intake, under-reporting of food intake is a limiting factor in self-reported questionnaires [36]. In order to improve the accuracy of the food descriptions, data entries were checked by a nutritionist and standard household measures were used. Another potential limitation is the major difference in sample size identified between anti-inflammatory diet group (*n* = 13) and pro-inflammatory diet group (*n* = 77). Finally, the DII score was calculated based on 28 of the 45 nutritional parameters of the original DII. The rest of the dietary components were not included due to their low self-reporting. Nevertheless, a previous study has indicated that their absence has no effect on DII scores, since these dietary components are not usually consumed by the population [37].

On the other hand, the main strength of the present study is that it is the first to investigate the relationship between the inflammatory potential of diet, as measured by the DII, and cardiometabolic risk parameters in a Latin American population. Since there have been no published studies conducted in populations of South and Central America, this could imply that our findings may not be generalizable to those reported in other populations with different dietary habits. Future population-based studies, and especially longitudinal studies, are required to evaluate the discriminatory power of the DII in Colombian subjects with different criteria and cardiovascular markers.

## 5. Conclusions

In summary, our results suggest that an increased anti-inflammatory potential of diet, as measured by the DII, was associated with an improved cardiometabolic profile. These findings underline the importance of promoting anti-inflammatory nutrition as an effective strategy for preventing CVD.

## Figures and Tables

**Table 1:** Anthropometric characteristics, body composition, metabolic biomarkers, and endothelial function variables according to the categories of the DII.

Characteristic	Anti-Inflammatory Diet (*n* = 13)	Pro-Inflammatory Diet (*n* = 77)	*p* Value
*Anthropometry*			
Age (years)	39.2 (7.2)	39.8 (6.9)	0.756
Weight (kg)	78.6 (13.7)	79.9 (11.6)	0.497
Height (cm)	164.3 (9.5)	162.4 (7.7)	0.206
Waist circumference (cm)	90.3 (10.1)	92.7 (9.3)	0.815
Hip circumference (cm)	105.9 (6.7)	106.5 (8.2)	0.327
Body mass index (kg/m²)	28.9 (2.8)	30.2 (3.6)	0.245
*Nutritional state*			
Overweight *n* (%)	8 (61.5)	41 (53.2)	0.714
Obesity *n* (%)	5 (38.5)	36 (46.8)	0.461
*Body composition*			
DXA trunk tissue (% fat)	41.4 (5.3)	43.7 (5.9)	0.437
DXA trunk lean (g)	21,913.1 (4418.1)	21,458.6 (3425.7)	0.105
DXA gynecoid tissue (% fat)	37.6 (8.7)	39.5 (8.7)	0.944
DXA gynecoid lean (g)	7405.9 (1909.5)	7087.2 (1307.0)	0.113
DXA android tissue (% fat)	44.0 (5.8)	46.3 (6.3)	0.424
DXA android lean (g)	3237.4 (662,9)	3196.8 (521.9)	0.393
DXA total tissue (% fat)	38.2 (6.2)	39.5 (6.5)	0.677
DXA total tissue (kg lean mass)	46.7 (10.6)	45.9 (8.0)	0.065
Quotient trunk/total	0.54 (0.05)	0.55 (0.06)	0.306
Quotient legs/total	0.32 (0.06)	0.31 (0.06)	0.598
Quotient arms + legs/trunk	0.80 (0.19)	0.77 (0.21)	0.561
Quotient tissue android/gynecoid	1.20 (0.24)	1.20 (0.24)	0.948
Appendicular index (kg/m²)	7.7 (1.2)	7.9 (1.1)	0.394
Fat-free lean mass index (kg/m²)	10.5 (2.4)	11.5 (3.0)	0.380
*Metabolic markers*			
TC (mg/dL)	162.8 (30.2)	159.2 (38.1)	0.373
Triglycerides (mg/dL)	158.9 (87.8)	194.5 (121.8)	0.379
HDL-C (mg/dL)	38.7 (9.2)	31.8 (5.1)	0.010
LDL-C (mg/dL)	99.8 (33.7)	94.1 (27.9)	0.372
Glucose (mg/dL)	87.7 (8.1)	92.5 (11.9)	0.070
MetScore	−0.176 (2.783)	1.812 (2.356)	0.017
HbAc1 (%)	5.3 (0.5)	5.8 (0.4)	0.003
*Markers of endothelial function*			
FMD (%)	12.3 (7.9)	6.7 (5.4)	0.015
Baseline diameter, mm	3.2 (0.5)	3.1 (0.3)	0.490
Reactive hyperemia diameter, mm	3.6 (0.7)	3.3 (0.4)	0.029
Baseline flow velocity, cm/s	80.4 (24.5)	81.9 (24.0)	0.836
Reactive hyperemia diameter, flow velocity, cm/s	152.8 (33.5)	135.3 (35.2)	0.107
Peak Shear rate, s	483.2 (118.0)	423.6 (99.9)	0.054
PWV (m/s)	7.0 (0.6)	7.3 (1.1)	0.082
Aortic systolic pressure (mm Hg)	110.7 (6.8)	111.0 (11.2)	0.889
Aortic pulse pressure (mm Hg)	39.1 (6.3)	38.8 (7.4)	0.886
Brachial augmentation index (%)	20.6 (11.4)	23.7 (19.0)	0.435
Aortic augmentation index (%)	−33.6 (22.4)	−20.8 (27.2)	0.082
*Blood pressure*			
SBP (mm Hg)	115.3 (9.7)	117.5 (7.5)	0.335
DBP (mm Hg)	71.5 (7.2)	72.2 (9.7)	0.767
MAP (mm Hg)	87.0 (6.5)	86.6 (9.2)	0.884

Data are shown as mean ± SD or frequencies *n* (%). The categories of the DII were compared using Student’s *t*-tests for continuous variables and chi-squared tests for categorical variables. DXA: Dual energy X-ray absorptiometry; TC: total cholesterol; c-HDL-C: high-density lipoprotein-cholesterol; LDL-C: low-density lipoprotein cholesterol; Hb1Ac: glycated hemoglobin; FMD: flow-mediated vasodilation; PWV: pulse wave velocity; SBP: systolic blood pressure; DBP: diastolic blood pressure; MAP: mean arterial pressure. The MetScore was calculated from the sum of the typified residuals (Z) of cardiovascular risk variables: ((♂40 or ♀50 − HDL-C)/SD × (−1))) + ((triglycerides − 150)/SD) + ((glucose − 100)/SD) + ((CC − ♂94 or ♀80)/SD) + ((MAP − 100)/SD, adjusted for gender and age.

**Table 2:** Nutrient intake according to the categories of the DII.

Nutrients	Anti-Inflammatory Diet (*n* = 13)	Pro-Inflammatory Diet (*n* = 77)	*p* Value
Energy (kcal)	2043.7(656.1)	1820.3 (602.4)	0.239
Protein (g)	85.1 (33.8)	74.4 (27.2)	0.219
Total fat (g)	81.8 (34.1)	72.8 (23.5)	0.252
Carbohydrate (g)	265.2 (149.4)	214.4 (86.6)	0.093
Fiber (g)	42.9 (50.1)	14.7 (6.4)	<0.000
Iron (mg)	30.1 (32.9)	14.4 (9.3)	0.001
Zinc (mg)	13.2 (4.5)	8.9 (5.2)	0.008
Magnesium (mg)	896.5 (699.8)	462.5 (575.8)	0.020
Vitamin A (RE)	1183.1 (946.9)	657.1 (647.3)	0.016
Vitamin C (mg)	146.6 (56.2)	69.6 (58.3)	<0.000
Thiamin (mg)	5.0 (5.4)	1.7 (1.3)	<0.000
Riboflavin (mg)	3.2 (4.9)	1.2 (0.5 )	<0.000
Niacin (mg)	28.2 (28.6)	15.3 (6.3)	0.001
Saturated fatty acid (g)	19.6 (10.3)	19.0 (9.1)	0.841
Mono-unsaturated fatty acid (g)	23.7 (17.0)	18.3 (8.2)	0.080
Polyunsaturated fatty acid (g)	39.1 (75.1)	19.2 (29.5)	0.096
Cholesterol (mg)	178.9 (112.4)	250.8 (199.8)	0.228
Trans fat (g)	0.2 (0.8)	0.4 (2.8)	0.854
Omega 6 (g)	33.7 (69.8)	21.1 (57.1)	0.492
Omega 3 (g)	2.3 (1.8)	1.8 (0.6)	0.046
Vitamin B6 (mg)	9.7 (21.6)	0.9 (0.4)	<0.000
Selenium (μg)	59.3 (35.6)	56.0 (44.3)	0.806
Folic acid (mg)	951.5 (2283.9)	34.9 (57.6)	<0.000
Vitamin B12 (μg)	11.6 (23.2)	2.5 (1.91)	0.001
Beta Carotene (μg)	2824.8 (2195.1)	1458.7 (1986.2)	0.031
Vitamin E (mg)	7.5 (4.5)	3.9 (2.2)	<0.000
Vitamin D (μg)	4.8 (7.5)	2.5 (2.0)	0.028
Caffeine (g)	0.7 (2.3)	0.2 (1.2)	0.299

Data are shown as mean (SD).

**Table 3:** Partial correlations between percentage of fat, metabolic markers, and endothelial function according to the categories of the DII.

Characteristic	Anti-Inflammatory Diet (*n* = 13)	Pro-Inflammatory Diet (*n* = 77)
DXA total tissue (% fat)	−0.122	0.111
TC (mg/dL)	−0.210	0.010
Triglycerides (mg/dL)	−0.354 *	−0.009
HDL-C (mg/dL)	−0.100	0.028
LDL-C (mg/dL)	0.350	−0.084
Glucose (mg/dL)	−0.422 *	−0.228
MetScore	−0.282 *	0.410 *
HbAc1 (%)	0.004	0.090
FMDn (%)	0.261 *	−0.233 *
PWV (m/s)	−0.437 *	0.014
Aortic systolic pressure (mm Hg)	−0.271	−0.126
Aortic pulse pressure (mm Hg)	−0.271	−0.055
Brachial augmentation index (%)	−0.300	−0.209
Aortic augmentation index (%)	−0.299	−0.064
MAP (mm Hg)	−0.011	0.079

* *p* < 0.05 through Pearson’s correlation. TC: total cholesterol; HDL-C: high-density lipoprotein-cholesterol; LDL-C: low-density lipoprotein cholesterol; Hb1Ac: glycated hemoglobin; FMD: flow-mediated vasodilation; PWV: pulse wave velocity; MAP: mean arterial pressure. The MetScore was calculated from the sum of the typified residuals (Z) of cardiovascular risk variables: ((♂40 or ♀50 − HDL-C)/SD × (−1)) + ((triglycerides − 150)/SD) + ((glucose − 100)/SD) + ((CC − ♂94 or ♀80)/SD) + ((MAP − 100)/SD, adjusted for gender and age.

## Abbreviations

BMI	Body mass index
CVD	Cardiovascular disease
DBP	Diastolic blood pressure
DII	Dietary inflammatory index
DXA	Dual-energy X-ray absorptiometry
FMD	Flow-mediated dilation
Hb1Ac	Glycosylated hemoglobin
HC	Hip circumference
HDL-C	High-density lipoprotein-cholesterol
LDL-C	Low-density lipoprotein-cholesterol
MAP	Mean arterial pressure
MetScore	Cardiometabolic risk score
PWV	Pulse wave velocity
SBP	Systolic blood pressure
TC	Total cholesterol
TG	Triglycerides
WC	Waist circumference
